# Short‐Term Effects of Meteorological Factors on Severe Fever With Thrombocytopenia Syndrome Incidence in Xinyang, China

**DOI:** 10.1029/2025GH001440

**Published:** 2025-08-04

**Authors:** Quanman Hu, Yan Hu, Yanyan Yang, Jundong Chen, Songshan Zhang, Fei Zhao, Saiwei Lu, Li Zhang, Shuaiyin Chen, Guangcai Duan

**Affiliations:** ^1^ College of Public Health Zhengzhou University Zhengzhou China; ^2^ Disease control and prevention center Xinyang China; ^3^ Disease control and prevention center Shangcai China

**Keywords:** severe fever with thrombocytopenia syndrome, incidence, meteorological factors, short term

## Abstract

Severe fever with thrombocytopenia syndrome (SFTS) is a tick‐borne zoonotic disease, which are classified by the World Health Organization as a priority disease for research and development in emergency situations due to the high mortality rate. Previous studies indicated that the complex nonlinear and delayed association was observed between meteorological factors and SFTS. However, these did not consider the short‐term effect of meteorological factors on the incidence of SFTS. In this study, we used generalized additive models (GAM) and distributed lag nonlinear models (DLNM) to investigate the short‐term correlation between meteorological factors and SFTS incidence. From 1 January 2013 to 31 December 2023 a total of 6,601 cases of SFTS were reported in Xinyang. Females constituted the majority with a male‐to‐female ratio of 0.68 and the average age of cases being approximately at around 61.52 years old. The multivariate GAM analysis revealed that mean temperature exerted the greatest influence on the incidence of SFTS compared to other meteorological factors and interacted with these factors. After accounting for lag period of 0–14 days, the DLNM analysis indicated that specific range of temperature (18–23°C), a certain range atmospheric pressure (1,006–1,017 hPa), extreme high wind speed (>11.6 m/s), and prolonged sunshine duration (>9h) were associated with SFTS, while there was no significant correlation between relative humidity and the incidence of SFTS. This study investigates the non‐linear trend and lagged exposure effect of various meteorological factors on short‐term SFTS incidence, thereby enhancing our comprehensive understanding of the effect of meteorological factors on SFTS.

## Key Points

- A total of 6,601 cases of Severe fever with thrombocytopenia syndrome (SFTS) were reported from 1 January 2013 to 31 December 2023 in Xinyang, China
- Generalized additive models and distributed lag nonlinear models were used to investigate the short‐term correlation between meteorological factors and SFTS incidence
- Temperature (18–23°C), amospheric pressure (1,006–1,017 hPa), wind speed (>11.6 m/s), and sunshine duration (>9h) were associated with SFTS incidence in the short term

## Graphical abstract

From 1 January 2013 to 31 December 2023 a total of 6,601 cases of SFTS were reported in Xinyang. The incidence of SFTS exhibits evident seasonality, commencing from March and gradually escalating from April, peaking between May and June, followed by a decline with reduced incidence after October. To investigate the short‐term correlation between meteorological factors and SFTS incidence, generalized additive models (GAM) and distributed lag nonlinear models (DLNM) were employed for evaluation. The results indicated that specific temperature ranges (18–23°C), certain atmospheric pressure ranges (1,006–1,017 hPa), extremely high wind speeds (>11.6 m/s), and prolonged sunshine durations (>9h) increase the short‐term risk of SFTS.

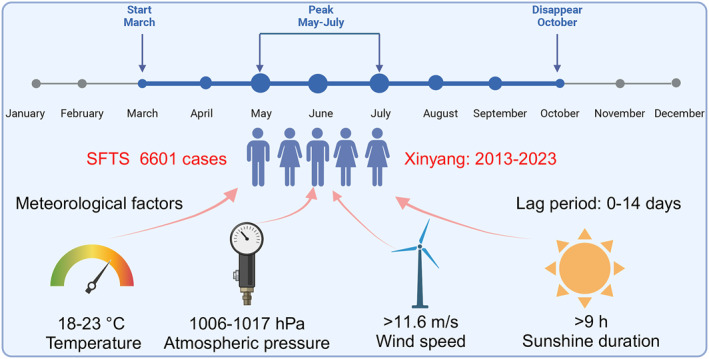

## 1 Introduction

Severe fever with thrombocytopenia syndrome (SFTS) is a tick‐borne zoonotic disease characterized by an abrupt onset of fever, accompanied by respiratory or gastrointestinal symptoms, followed by progressive decline in platelet count and white blood cell levels (Li et al., 2018; Liu et al., 2014). The first detection of SFTS occurred in China in 2009 (Yu et al., 2011), and subsequent cases have been reported in other countries including South Korea (Yun et al., 2014), Japan (Takahashi et al., 2014), and Vietnam (Tran et al., 2019). Through metagenomic analysis, it was determined that the infection was caused by a novel Bunyavirus (SFTSV) (Xu et al., 2011). The SFTSV, similar to other members within the Bunyaviridae family, is an enveloped RNA virus characterized by three single‐stranded RNA genomes comprising of large (L), medium (M), and small (S) segments (Li, 2013). In recent years, China has continued to report SFTS cases with a high fatality rate, which has aroused great attention from the China Centers for Disease Control and Prevention (CDC) and the government. In 2018, it was listed as a priority disease for research and development in emergency situations by the World Health Organization (Li et al., 2021).

SFTS is primarily transmitted through ticks, and Haemaphysalis longicornis is one of the primary vectors of SFTSV in East Asia (Jung et al., 2019; Zhuang et al., 2018). Infected ticks can transmit the virus to humans and other host animals during feeding, thereby influencing the transmission dynamics and prevalence of SFTS (Jiao et al., 2015; Jung et al., 2019; Luo et al., 2015). Previous studies have demonstrated a strong correlation between the incidence of SFTS and the geographical environment. Specifically, SFTS cases are predominantly observed in regions characterized by temperate humid or subtropical climates (Miao et al., 2021). And it has been noted that most affected individuals are farmers residing in wooded and hilly areas (Du et al., 2014; D. Zhang et al., 2019). The possible reason may be attributed to the fact that forest fragmentation and climate warming enhance the likelihood of SFTSV infection in humans (Iijima et al., 2025). Several studies have employed an ecological niche modeling method to identify key factors influencing SFTS occurrence, revealing significant associations with meteorological variables such as altitude, annual mean temperature, annual cumulative precipitation, and annual mean relative humidity (Du et al., 2014; J. M. Sun et al., 2021).

At the same time, the comprehensive results from the generalized additive model (GAM) and the generalized cross‐validation score (GCV) indicated that sunshine duration, relative humidity, temperature, and tick density were critical factors influencing the occurrence of SFTS. However, these analyses did not account for potential hysteresis effects (Deng et al., 2022). Furthermore, a complex nonlinear and delayed association was observed between meteorological factors and the incidence of SFTS. Jiang et al., utilizing the GAM, identified an inverted “V” relationship between mean temperature, precipitation, forest and grassland coverage, altitude, and estimated SFTS cases (Jiang et al., 2022). Subsequent study revealed that meteorological factors such as temperature and sunshine duration exerted an influence on morbidity with a prolonged lag time (Wang et al., 2024).

However, these studies did not consider the short‐term effect of meteorological factors on the incidence of SFTS. According to the 2023 edition of China's diagnosis and treatment protocol for SFTS, the incubation period for SFTS can range from 1 to 2 weeks, while in cases of human‐to‐human transmission, it is typically 6–9 days (Diagnosis and treatment of fever with thrombocytopenia syndrome (2023)), and being blood relatives of the index case, direct blood/bloody secretion contact and bloody droplet contact had more risk of infection (Fang et al., 2021). Previous studies have demonstrated that meteorological factors have a remarkable short effect on infectious diseases, such as hand, foot and mouth disease (Yang et al., 2024; Q. Zhang et al., 2019), epidemic hemorrhagic fever with renal syndrome (HFRS) (Luo et al., 2022), bacillary dysentery (Chang et al., 2022), and malaria (Guo et al., 2015). Xinyang is situated in the transitional zone between the subtropical and warm temperate zones, exhibiting a typical monsoon climate. It serves as the initial location in China where SFTS cases were identified, demonstrating a high incidence rate. In this study, we used GAM and distributed lag nonlinear models (DLNM) to investigate the short‐term correlation between meteorological factors and SFTS incidence, which will provide novel insights into the epidemiological evidence concerning the transmission of SFTS.

## 2 Methods

### 2.1 Ethics Statement

This study has been approved from the Life Science Ethics Review Committee of Zhengzhou University (ZZUIRB2024‐205). In accordance with the Law of the People's Republic of China on the Prevention and Control of Infectious Diseases, it is obligatory to report demographic and disease information pertaining to SFTS, therefore, written consent is not required.

### 2.2 Study Area

**Figure 1:**
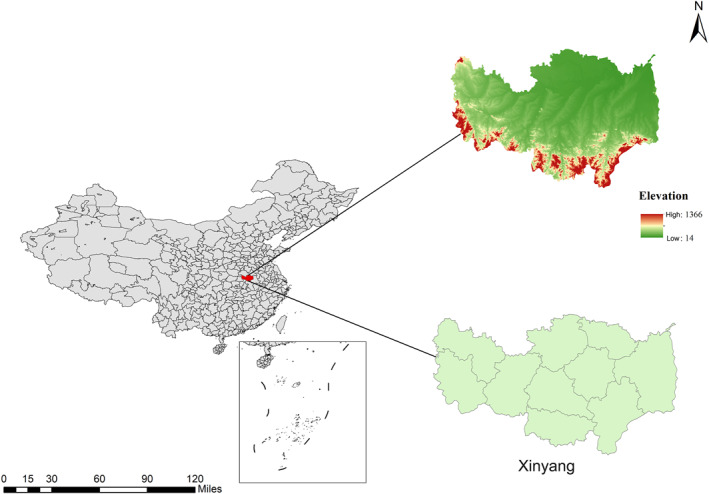
The location of the study area in China. The left figure shows a map of China, with Xinyang city in red; The upper right is the terrain map of Xinyang, and the lower right is the administrative division of Xinyang. The base layer of the map is from the China National Platform for Common GeoSpatial Information Services (www.tianditu.gov.cn).

Xinyang is situated in Henan Province, China, at coordinates 32°03'N and 114°08'E. It boasts a population of approximately 6,048,800 inhabitants and covers an area spanning over 8,916 square kilometers. Geographically speaking, Xinyang exhibits a topography characterized by higher elevations in the south gradually descending toward lower altitudes in the north (Figure 1). Furthermore, it resides within the transitional zone between the subtropical and warm temperate climatic regions, making it representative of a typical monsoon climate with an annual average temperature ranging from 15.3 to 15.8°C and precipitation levels varying between 993 and 1,294 mm.

### 2.3 Data Collection

According to the Chinese law and Diagnosis and treatment of fever with thrombocytopenia syndrome (2023 edition), SFTS cases must be reported to China Information System for Diseases Control and Prevention (CISDCP) within 24 hr of diagnosis. In Xinyang city, 80% of SFTS cases are directly diagnosed/reported by county‐level hospitals and designated SFTS hospitals with PCR testing capacity, and 20% of SFTS cases are initially flagged by township hospitals as suspected cases, then referred to higher‐level hospitals for further diagnosis. The final step is data validation: County CDCs audit reports; municipal CDCs conduct PCR verification to exclude false positives. The SFTS case data in Xinyang were obtained from the CISDCP covering the period from January 2013 to December 2023. The data set includes gender, age, address, occupation, date of onset, and date of diagnosis. The meteorological data from 2013 to 2023 of Xinyang in the same period were collected from the European Center for Medium‐Range Weather Forecasts (ECMWF) fifth‐generation global atmospheric reanalysis (ERA5) (Hersbach et al., 2023). The data primarily encompasses mean temperature (°C), mean atmospheric pressure (hPa), mean relative humidity (%), mean wind speed (m/s), and sunshine duration (h). Sunshine duration were defined as the sum of that sub‐period for which the direct solar irradiance exceeded 120 W/m m^2^ per day.

### 2.4 Statistical Analysis

The time trend of SFTS cases from 2013 to 2023 in Xinyang City and meteorological factors such as mean temperature (°C), mean atmospheric pressure (hPa), mean relative humidity (%), mean wind speed (m/s) and sunshine duration (h) were descriptive analyzed. The 0th, 25th, 50th, 75th and 100th percentiles were used to describe, and time series maps of cases with meteorological factors and associated bubble maps were drawn.

We used a log‐linear GAM analysis to investigate the association between meteorological factors and SFTS cases. In order to evaluate the multicollinearity among meteorological factors, Pearson correlation coefficient and Variance Inflation Factor (VIF) were used for diagnosis in this study. A correlation coefficient of <0.7 or 0 < VIF < 5 between two independent variables is considered to indicate no multicollinearity (Marcoulides & Raykov, 2019; Waldmann, 2019).

A model was constructed linking the expected logarithm of SFTS cases to meteorological variables, assuming that daily SFTS cases approximately follow a quasi‐Poisson distribution. The optimal degree of freedom (df) for the spline function was estimated using GCV criteria. By incorporating time trends as confounding factors, the model was calibrated and defined as follows:

$$\mathrm{Log}\;Y_{t}=\alpha +s\;\left(MF_{t},\text{df}\right)+s\;\left(\text{Day},\text{df}\right)$$

where, log (Yt) represents logarithmic conversion of the number of SFTS cases on day_t_. α is the intercept. MF_t_ is the meteorological factor of day_t_. s () refers to the thin plate spline function based on penalty smoothing splines. df is the degree of freedom.

The nonlinear and delayed association between various meteorological factors and SFTS cases was quantified using a quasi‐Poisson regression model combined with DLNM sequence (Gasparrini, 2011; Mingrone et al., 2016). The formulated model is as follows:

$$\mathrm{Log}\;E\left(Y_{t}\right)=\alpha +\text{cv}\left(M\right)+\text{ns}\left(\text{mete},\text{df}\right)+\text{ns}\;\left(t\text{ime},\text{df}\right)+\beta \;\text{DOW}$$

The number of cases of SFTS, denoted as Y, is modeled using the expected value E(*Y*_t_). The cross‐basis matrix cv(M) is employed to study various meteorological factors represented by M ns (mete, df) is a nonlinear smooth term that employs natural cubic splines with df degrees of freedom to model meteorological variables (mete). Based on the Quasi‐AIC criterion, the final degrees of freedom are set at 4 and 3 respectively. The median value of different meteorological factors serves as the reference point, while a lag period of up to 14 days is considered based on research consensus from China CDC (Diagnosis and treatment of fever with thrombocytopenia syndrome (2023)). Time variable is included to control for long‐term trends and seasonality with an annual degree of freedom set at eight in this model. Dow represents the week effect used for adjusting weekday and non‐weekday effects. The relative risk (RR) is calculated for varying values of each meteorological factor and for each lag (0–14 days), using the crosspred () function with a median reference value as the baseline. The cumulative (cum) RR and 95% confidence interval (CI) are calculated for varying values of each meteorological factor and for cumulative lag 0–14 days, using the crossreduce () function with a median reference value as the baseline.

When investigating extreme meteorological factors such as extreme high and low pressure, the reference values are defined as the 5th and 95th percentiles respectively. Effects (RR and 95% CI) for each exposure factor are calculated daily within a range from lag 0–14 days: using the crosspred () function, specify the data for meteorological factors (5th/95th), the median reference value.

DLNM Model goodness of Fit test: the explanatory power of the model is assessed by calculating McFadden's pseudo R‐squared (R^2^), and the effect of the “time” and “DOW” variables is removed using ANOVA.

### 2.5 Sensitivity Analysis

The stability of the model was assessed by varying the annual degrees of freedom for time in the GAM model (ranging from 8 to 12) and DLNM model (ranging from 7 to 9), and exploring different maximum lag times (7, 14, 21 days) to assess the stability of DLNM model. The statistical analysis software used in this study was R4.3.3, and we primarily utilized packages such as “dlnm,” “tsModel,” “splines,” and “openair” to construct our model. All statistical analysis were two‐sided, with a significance level set at *P* < 0.05.

## 3 Results

### 3.1 Basic Characteristics of SFTS Cases and Meteorological Factors

From 1 January 2013 to 31 December 2023, a total of 6,601 cases of SFTS were reported in Xinyang City. It is worth noting that the annual case of SFTS in 2015 and 2016 was the highest, with 1,028 and 868 cases respectively. The demographic data of SFTS cases were showed in Table S1 in Supporting Information S1 and the meteorological data are presented in Table S2 in Supporting Information S1.

**Figure 2:**
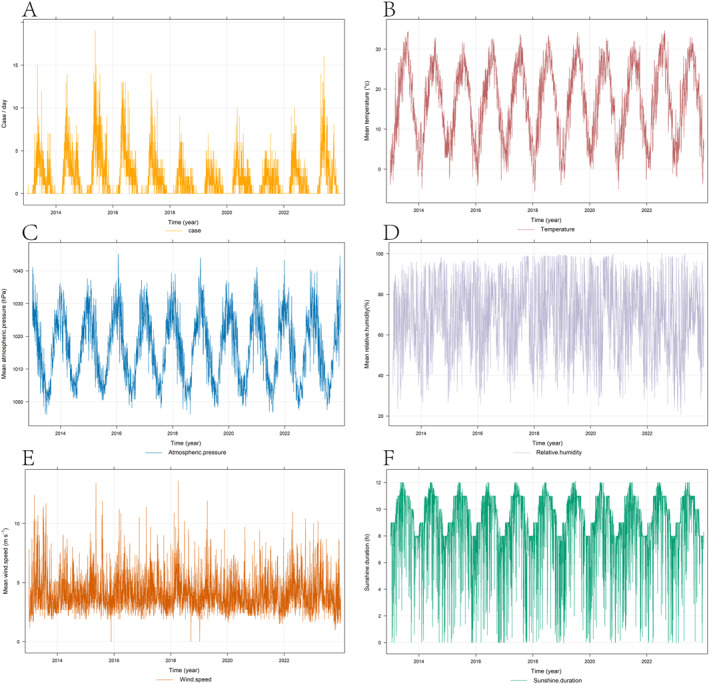
Time‐series regarding the association of daily Severe fever with thrombocytopenia syndrome cases with meteorological factors in Xinyang from 2012 to 2019. (a) (case/day); (b) (Mean temperature); (c) (Mean atmospheric pressure); (d) (Mean relative humidity); (e) (Mean wind speed); (f) (Sunshine duration).

The temporal pattern of SFTS case/per day is depicted in Figure 2a. The incidence of SFTS exhibits evident seasonality, commencing from March and gradually escalating from April, peaking between May and June, followed by a decline with reduced incidence after October. Meteorological factors (mean temperature, mean atmospheric pressure, mean relative humidity, mean wind speed, and sunshine duration) also exhibit significant periodicity with relatively stable patterns (Figures 2b–2e).

### 3.2 Association Between Different Meteorological Factors and SFTS Incidence

**Figure 3:**
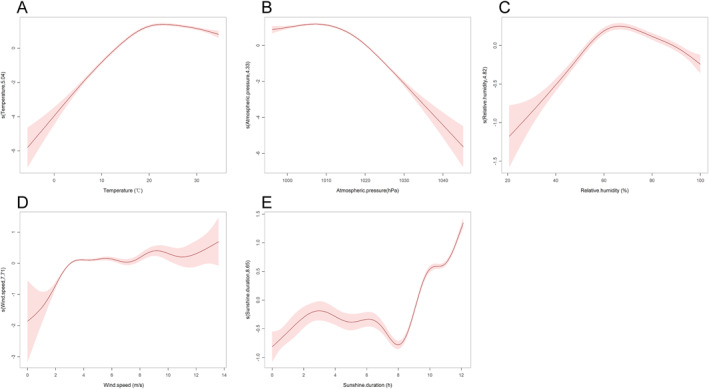
Exposure‐response curves for the effects of different meteorological factors on daily Severe fever with thrombocytopenia syndrome (SFTS) cases in the single‐variable model using univariate generalized additive models. (a) (Daily mean temperature); (b) (Daily mean atmospheric pressure); (c) (Daily mean relative humidity); (d) (Daily mean wind speed); (e) (Sunshine duration). The *x*‐axis is the meteorological parameters. The *y*‐axis indicates the contribution of the smoother to the fitted value. A *Y*‐axis value of 0 implies that the contribution of meteorological factors to the number of SFTS cases is at the baseline level. A *Y*‐axis value > 0 indicates a positive contribution of meteorological factors to the number of SFTS cases, meaning an increased risk of incidence. Conversely, a *Y*‐axis value < 0 indicates the opposite situation. S (Temperature, 5.04), where 5.04 represents the effective degree of freedom of the smoothing function.

We used GAM to perform a univariate analysis of various meteorological factors. As shown in Figure 3 and Figure S1 in Supporting Information S1, When the mean temperature exceeds 13°C, the risk of SFTS increases in tandem with the rise in the mean temperature. It reaches a peak at 22.8°C and then starts to decline. Similarly, a decrease in mean atmospheric pressure below 1,020.2 hPa corresponds to an increase in the risk of SFTS as the mean atmospheric pressure rises. This risk peaks at 1,007.3 hPa before declining. The mean relative humidity levels associated with these conditions range from 53.6% to 88.1%, with a peak value observed at 67.7%. Moreover, when the mean wind speed exceeds 3.1 m/s or the sunshine duration surpasses 9.2 hr, the risk of SFTS rises in tandem with these variables' increments. At the same time, we also examined the relationships between minimum and maximum temperatures and the incidence of SFTS. We found that these trends were similar to those observed for mean temperature; however, discrepancies existed in the peak temperatures (Figure S2 in Supporting Information S1).

**Figure 4:**
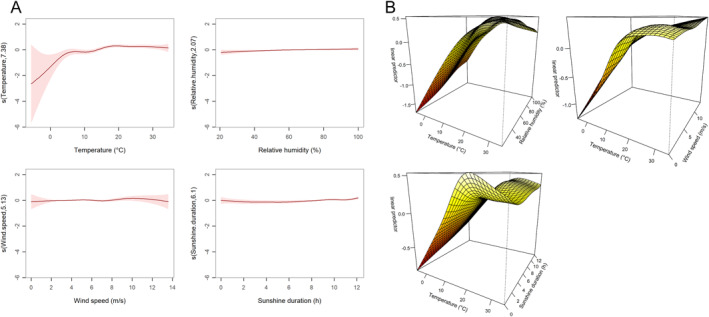
Exposure‐response curves and the interactive effects for the effects of different meteorological factors on daily Severe fever with thrombocytopenia syndrome (SFTS) cases in the multiple‐variable model using multivariable generalized additive models. (a) Exposure‐response curves. The *x*‐axis is the meteorological parameters. The *y*‐axis indicates the contribution of the smoother to the fitted value. A *Y*‐axis value of 0 implies that the contribution of meteorological factors to the number of SFTS cases is at the baseline level. A *Y*‐axis value > 0 indicates a positive contribution of meteorological factors to the number of SFTS cases, meaning an increased risk of incidence. Conversely, a *Y*‐axis value < 0 indicates the opposite situation. S (Temperature,7.38), where 7.38 represents the effective degree of freedom of the smoothing function. (b) The interactive effects: (“mean temperature pressure”‐“relative humidity”; “mean temperature pressure”‐“wind speed”; “mean temperature pressure”‐“sunshine duration”). The *Y*‐axis (linear predictor) depicts the number of predicted SFTS cases on a logarithmic scale. It should be noted that the actual number of cases must be exponentially converted.

Subsequently, we conducted collinearity analysis among these meteorological factors and identified a high degree of collinearity between mean temperature and mean atmospheric pressure, with VIF of 5.848 and 5.662, respectively. The spearman correlation coefficient between the two meteorological factors was −0.908 (*P* < 0.001). Given this strong collinearity, mean atmospheric pressure was excluded prior to the multivariate GAM analysis. The multivariate GAM analysis revealed that mean relative humidity and mean wind speed had a relatively weaker effect on the incidence of SFTS compared to mean temperature and sunshine duration (Table S3 in Supporting Information S1, Figure 4a). Meanwhile, the interactions between mean temperature and mean relative humidity, mean wind speed and sunshine duration, were all significant and complex (Table S3 in Supporting Information S1, Figure 4b).

### 3.3 Comprehensive Effects of Different Meteorological Factors on the Nonlinearity and Delayed of SFTS Incidence

**Figure 5:**
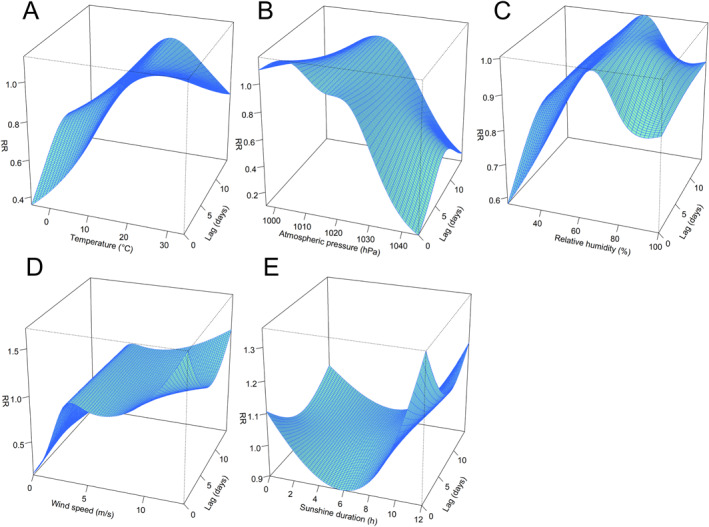
The estimated exposure‐lag‐response 3D plots of meteorological factors. (a) (Daily mean temperature); (b) (Daily mean atmospheric pressure); (c) (Daily mean relative humidity); (d) (Daily mean wind speed); (e) (Sunshine duration).

Then we used DLNM model to evaluate the hysteresis and nonlinear effects of various meteorological factors,and the 3‐D plots can describe the degree of association in terms of exposure and hysteresis (Figure 5). Table S4 in Supporting Information S1 describes the contributions of “time” and “dow” to the model. In the models involving other meteorological factors except mean temperature, removing the “time” and “dow” variables did not result in a substantial reduction in model fit. For mean temperature, when the mean temperature is above 18°C, a shorter lag period is associated with a higher RR value (Figure 5a). Regarding mean atmospheric pressure exhibited variations, when the mean atmospheric pressure ranged from 997 to 1,014 hPa, the lower the lag days, the higher the RR of the incidence of SFTS (Figure 5b). While the daily mean relative humidity was not found to have a remarkable RR for SFTS incidence (Figure 5c). As for wind speed, a higher wind speed above 8.6 m/s coupled with a shorter lag day resulted in an increased RR (Figure 5d). Sunshine duration is different from other factors, when the sunshine duration exceeds 9 hr, both shorter and longer lag days are associated with higher RR values for SFTS risk compared to a lag of 8 days (Figure 5e).

**Figure 6:**
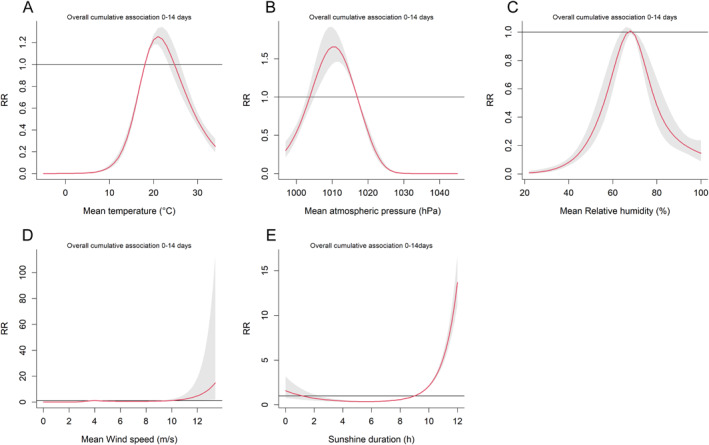
The overall cumulative‐response curves (relative risk and 95% confidence interval) of different meteorological factors and daily Severe fever with thrombocytopenia syndrome cases at lag 0–14 days. (a) (Daily mean temperature); (b) (Daily mean atmospheric pressure); (c) (Daily mean relative humidity); (d) (Daily mean wind speed); (e) (Daily sunshine duration). The reference for all estimates was the median of each meteorological factor.

Subsequently, we analysis the cumulative (cum) effect of SFTS incidence on 0–14 lag days of different meteorological factors. As shown in Figure 6 and Table S5 in Supporting Information S1, cum RR and 95% CI of mean temperature were consistently above 1 starting from 18 to 23°C; corresponding mean atmospheric pressure: 1,006–1,017 hPa; corresponding mean wind speed: >11.6 m/s; corresponding sunshine duration: >9 hr. However, for mean relative humidity, no cum RR and 95% CI was observed to exceed 1 on 0–14 lag days.

### 3.4 Effects of Different Extreme Meteorological Factors on the Hysteresis of SFTS Incidence

**Figure 7:**
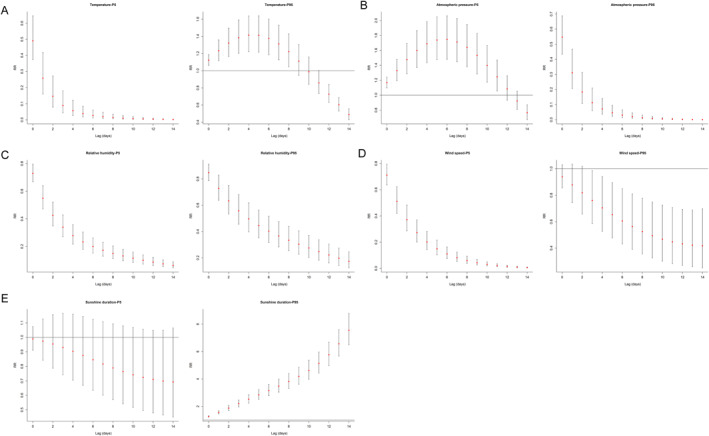
Effect of different extreme low (5th) and extreme high (95th) meteorological factors on daily Severe fever with thrombocytopenia syndrome across different lag periods (0–14 days). (a) (Daily mean temperature); (b) (Daily mean atmospheric pressure); (c) (Daily mean relative humidity); (d) (Daily mean wind speed); (e) (Daily sunshine duration). The reference for all estimates was the median of each meteorological factor.

We examined the RR and 95%CI for different lag days under extreme low (5th percentile) and extreme high (95th percentile) mean meteorological factors. For mean temperature, we observed that the RR and 95% CI for extreme low mean temperature consistently remained below 1 across all lag days. In contrast, the RR and 95% CI for extreme high mean temperature were above 1 at lag 9 days, with a peak occurring at lag 4 days (RR = 1.415, 95% CI: 1.222–1.639) (Figure 7a, Table S6 in Supporting Information S1). For atmospheric pressure, the RR and 95% CI for extreme low mean pressure reached their peak at lag 6 days (RR = 1.747, 95% CI: 1.480–2.063), whereas the RR and 95% CI for extreme high mean pressure were all below 1 (Figure 7b, Table S7 in Supporting Information S1). Additionally, the RR and 95% CI for both extreme low and high mean relative humidity were consistently below 1 (Figure 7c, Table S8 in Supporting Information S1). Subsequently, we analyzed extreme low and high wind speeds across various lag days (0–14 days) but found no evidence suggesting an association between either extreme and SFTS within this timeframe (Figure 7d, Table S9 in Supporting Information S1). Furthermore, for sunshine duration, the RR was <1 and the 95% CI included 1 for extreme low sunshine duration at lag 0–14 days. Conversely, for extreme high sunshine duration, the RR and 95% CI were >1 when the lag day >0, with both increasing as the lag day increased (Figure 7e, Table S10 in Supporting Information S1).

### 3.5 Sensitivity Analysis

The stability of the model was assessed by varying the degrees of freedom for time in the GAM model (ranging from 8 to 12) and DLNM model (ranging from 7 to 9) and adjusting the maximum lag days (ranging from 7 to 21 days). As depicted in Figure S3 and S4 in Supporting Information S1, a similar outcome as described above was obtained.

## 4 Discussion

This study primarily investigated the effect of various meteorological factors on the risk of SFTS within a lag 14 days. The findings revealed significant correlations between specific ranges of high temperature, a certain range low atmospheric pressure, extreme high wind speed and prolonged sunshine duration with short‐term SFTS incidence, demonstrating non‐linear and delayed effects. Conversely, relative humidity exhibited no association with SFTS incidence. In contrast to previous studies focusing on long‐term meteorological factors and their relationship with the risk of SFTS, our study specifically examined the link between short‐term meteorological factors and SFTS.

Multiple studies have demonstrated that temperature is a crucial environmental factor influencing insect‐borne infectious diseases, including dengue (Li et al., 2020), malaria (Gizaw et al., 2024), and HFRS (W. Sun et al., 2021). However, the relationship between temperature and the incidence of SFTS is not straightforward or linear. In this study, preliminary time series analysis and bubble map revealed that the majority of SFTS cases occurred within a temperature range of 20–30°C, while occurrences at both low and high temperatures were less frequent. Further multivariate GAM analysis demonstrated that temperature exerted the greatest influence on the incidence of SFTS compared to other meteorological factors and interacted with these factors. This is primarily because temperature can directly affect the transmission of SFTSV(Yano et al., 1987). A meta‐analysis revealed that mean temperature exhibits the highest correlation coefficient with the incidence of SFTS among all meteorological factors (Mo et al., 2025). Additionally, Wang et al.'s study, which employed a random forest model, also identified temperature as the most critical meteorological factor influencing SFTS (Wang et al., 2024).

After accounting for lag period of 0–14 days, the DLNM analysis reveals that the risk of SFTS is significantly increased when temperatures range between 18 and 23°C.One possible explanation for these patterns is that extreme temperatures can suppress tick foraging activities (Ogden & Lindsay, 2016), while low temperatures induce hibernation in tick larvae, which only develop into nymphs when the temperature reaches a certain threshold again (Wang et al., 2021). Another study on Haemaphysalis longicornis indicates its temperature for oviposition, egg hatching (developmental zero), larval molting, and nymphal molting are 11.1°C, 12.2°C, 10.2°C, and 11.8°C, respectively (Yano et al., 1987). These factors may account for the absence of SFTS cases in the extremely low temperatures observed in this study. Meanwhile, the risk of SFTS decreases under high‐temperature conditions. This can be attributed to the reduced replication ability and adaptability of SFTSV at elevated temperatures but also to the fact that excessively high temperatures are detrimental to the survival of ticks (Feng et al., 2017; Loop et al., 2025; Sun et al., 2018).

The range of atmospheric pressure (1,006–1,017 hPa) is closely associated with the incidence of SFTS within a 0–14 lag days. This could be attributed to prolonged tick activity and reduced tick mortality resulting from low atmospheric pressure, which enhances nymphs' feeding success on their natural hosts and increases the likelihood of human‐tick contact (Li et al., 2012). A study conducted in Zhejiang, China, has also confirmed this: the probability risk of SFTS elevation initially exhibited an upward trend, peaked at approximately 400 m, and subsequently showed a downward trend (Tao et al., 2024). In contrast, the relationship between relative humidity and STFS was found to be non‐significant in a nonlinear manner. No increase in SFTS incidence was observed during both dry and wet weather conditions, contradicting the findings of a study conducted in Zhejiang, China (Wu et al., 2020). These discrepancies may arise due to variations in research methodologies employed. Our study primarily focuses on short‐term impacts of relative humidity on SFTS, while Wu et al.'s investigation emphasizes long‐term effects. Another contributing factor could be differences in climatic environments; Xinyang is situated inland with a temperate climate whereas Zhejiang has proximity to the ocean and experiences subtropical monsoon climate (Wu et al., 2020).

In addition, we only observed a significant correlation between extreme wind speed (>11.6 m/s) and SFTS cases. But Wang et al.'s study demonstrated a negative association between the incidence of SFTS and maximum gust velocity (Wang et al., 2024). Nevertheless, our findings suggest that the effect of wind speed on ticks might require longer exposure periods, as the lag of maximum wind speed in 14 days still yielded a 95% CI containing 1. Furthermore, prolonged sunshine duration was found to elevate the risk of SFTS. Consistent with Deng et al.'s results (Deng et al., 2022), when sunshine duration exceeded 10 hr, there was a sharp rise in the incidence of SFTS. Additionally, niche modeling indicated that continuous sunshine duration is a crucial environmental factor influencing SFTS transmission (Du et al., 2014). This could be attributed to factors such as increasing daylight hours promoting tick and host animal activity while simultaneously heightening human exposure probability to SFTSV (Ding et al., 2014).

In summary, specific range of temperature (18–23°C), a certain range low atmospheric pressure (1,006–1,017 hPa), extreme high wind speed (>11.6 m/s), and prolonged sunshine duration (>9h) were associated with SFTS incidence in the short term. However, further study is required to elucidate the underlying mechanisms involved. Our study possesses several strengths: (a) The study area is situated in central China within a transitional climate zone from temperate to subtropical regions compared to previous studies; (b) The case information has undergone repeated confirmation and laboratory monitoring using the earliest reported time after verification; (c) It focuses on investigating the short‐term effects of meteorological factors on SFTS for the first time while explaining their relationship with disease incidence over a brief period. Nevertheless, it should be noted that certain limitations exist: (a) The influence of rainfall on SFTS incidence was not considered due to minimal precipitation in Xinyang throughout most days of the year which was excluded from our DLNM model; (b) Extrapolation of research findings to other regions may be limited due to variations in climate and geographical conditions.

## 5 Conclusion

This study investigates the non‐linear trend and lagged exposure effect of various meteorological factors on short‐term SFTS incidence, thereby enhancing our comprehensive understanding of the effect of meteorological factors on SFTS. Moreover, we have proposed several recommendations for the CDC to enhance the control of SFTSV: (a) Optimize resource scheduling within specific weather conditions; (b) Integrate the key meteorological thresholds identified in this study into the existing CDC monitoring system and establish a weather‐risk‐related early warning mechanism.

**List of Abbreviations**
CDC: Centers for Disease Control and Prevention
CI: confidence interval
DLNM: distributed lag nonlinear models
GAM: generalized additive models
HFRS: hemorrhagic fever with renal syndrome
SFTS: severe fever with thrombocytopenia syndrome
SFTSV: a novel Bunyavirus

## Conflict of Interest

The authors declare no conflicts of interest relevant to this study.

## Supporting information

Supporting Information S1

## Data Availability

The SFTS case data were obtained from the information system of Xinyang CDC of Henan province, China. However, in compliance with China laws and policies that mandate the confidentiality of relevant patient information, the details of this data set will remain unpublished, and only the daily case data of SFTS in Xinyang from 2013 to 2023 were publicly available on Github (https://github.com/quanmanhu/DLNM‐code‐of‐one‐paper‐of‐Geohealth‐). The meteorological data of Xinyang in the same period were collected from the European Centre for Medium‐Range Weather Forecasts (ECMWF) fifth‐generation global atmospheric reanalysis (ERA5) (Hersbach et al., 2023). The base layer of the map is from the China National Platform for Common GeoSpatial Information Services (https://cloudcenter.tianditu.gov.cn/administrativeDivision/. Note: this page is displayed in Chinese by default. English readers may access the information by using the translation feature in browsers such as Edge.). Additionally, the code of R used to perform any data analysis and to produce the manuscript's figures were made available on Github (https://github.com/quanmanhu/DLNM‐code‐of‐one‐paper‐of‐Geohealth‐) and Zenodo (Hu, 2025).
