# Supplementary material for: Short‐Term Effects of Meteorological Factors on Severe Fever With Thrombocytopenia Syndrome Incidence in Xinyang, China
Source: Geohealth. 2025 Aug 4;9(8):e2025GH001440. doi: 10.1029/2025GH001440 (PMC12320122; doi:10.1029/2025GH001440)
Supplement: Supplementary file 1 — Supporting Information S1 [file GH2-9-e2025GH001440-s001.pdf]

GeoHealth

Supporting Information for

Short-term effects of meteorological factors on severe fever with thrombocytopenia syndrome incidence in Xinyang, China

Quanman Hu[1]; Yan Hu[2]; Yanyan Yang[2]; Jundong Chen[2]; Songshan Zhang[3]; Fei Zhao[1;] Saiwei Lu[1]; Li Zhang[2*]; Shuaiyin Chen[1*]; Guangcai Duan[1]

[1] Affiliation for author 1: College of Public Health, Zhengzhou University, Zhengzhou 450001, China; [2] Affiliation for author2: Disease control and prevention center, Xinyang 463600, China; [3] Affiliation for author3: Disease control and prevention center, Shangcai 463800, China

Contents of this file

Introduction
Figures S1 to S4
Tables S1 to S10

Introduction

This supporting information provides figures/tables that are referenced in the main manuscript.

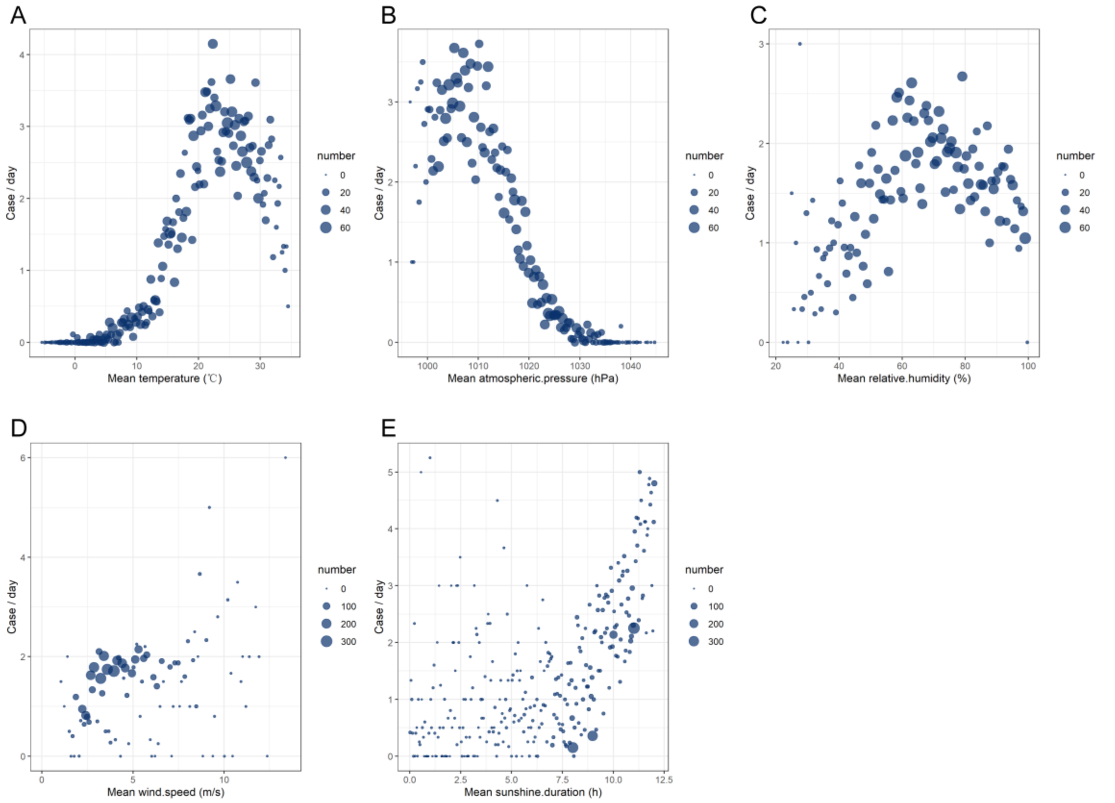

Figure S1 Bubble diagram regarding the association of SFTS incidence with meteorological factors in Xinyang from 2013 to 2023. A (Daily mean Temperature); B (Daily mean atmospheric pressure); C (Daily mean relative humidity); D (Daily mean wind speed); E (Daily sunshine duration)

Note: the vertical position of each point indicates the average daily case count across all days within the corresponding meteorological factor range. The size of each point represents the number of days included in the meteorological factor range.

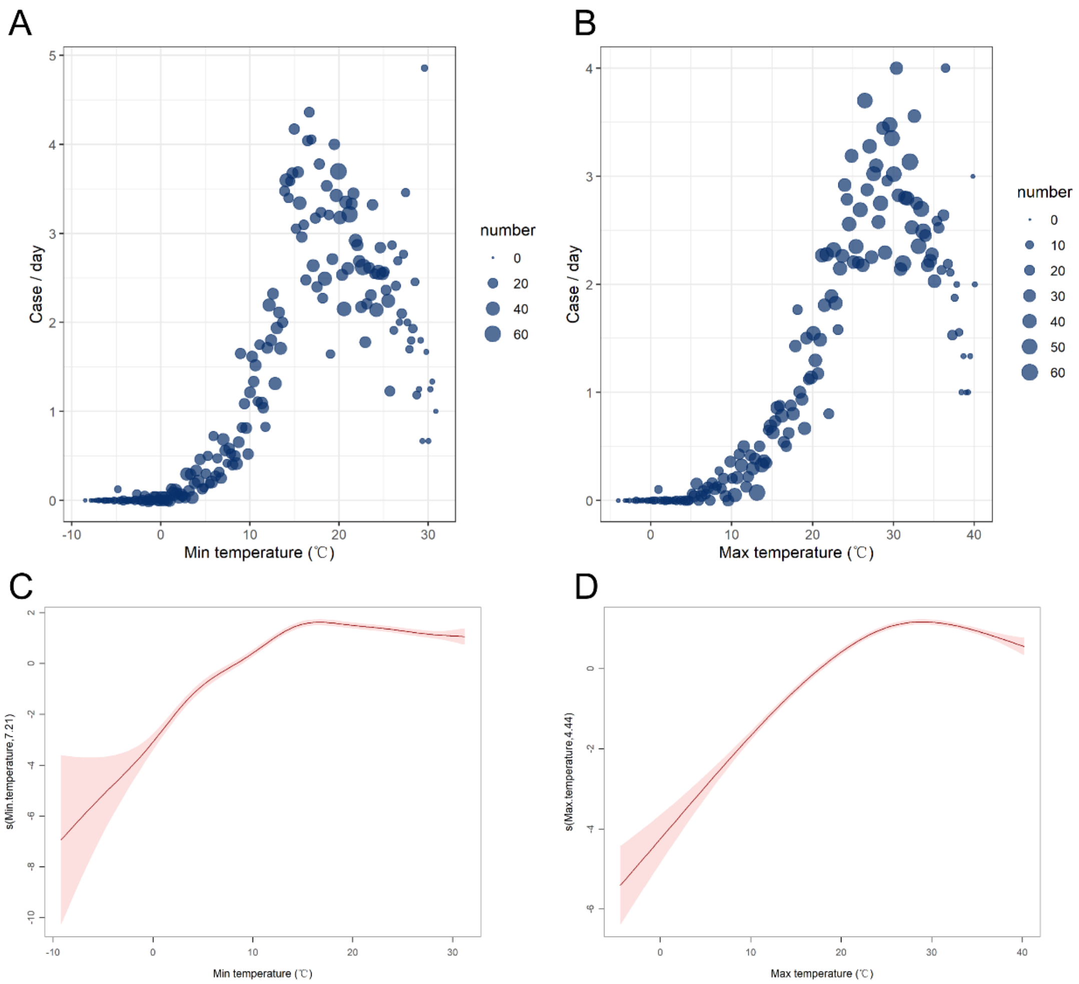

Figure S2 Bubble diagram and exposure-response curves for the effects of min temperature and max temperature on daily SFTS cases in the single-variable model using univariate GAM. A: Bubble diagram –daily min temperature; B: Bubble diagram –daily max temperature; C: Exposure-response curves –daily min temperature; D: Exposure-response curves –daily max temperature.

Note: A-B: the vertical position of each point indicates the average daily case count across all days within the corresponding meteorological factor range. The size of each point represents the number of days included in the meteorological factor range. C-D: The x-axis is the meteorological parameters. The y-axis indicates the contribution of the smoother to the fitted value. A Y-axis value of 0 implies that the contribution of meteorological factors to the number of SFTS cases is at the baseline level. A Y-axis value > 0 indicates a positive contribution of meteorological factors to the number of SFTS cases, meaning an increased risk of incidence. Conversely, a Y-axis value < 0 indicates the opposite situation. S (Temperature,7.21), where 7.21 represents the effective degree of freedom (EDF) of the smoothing function.

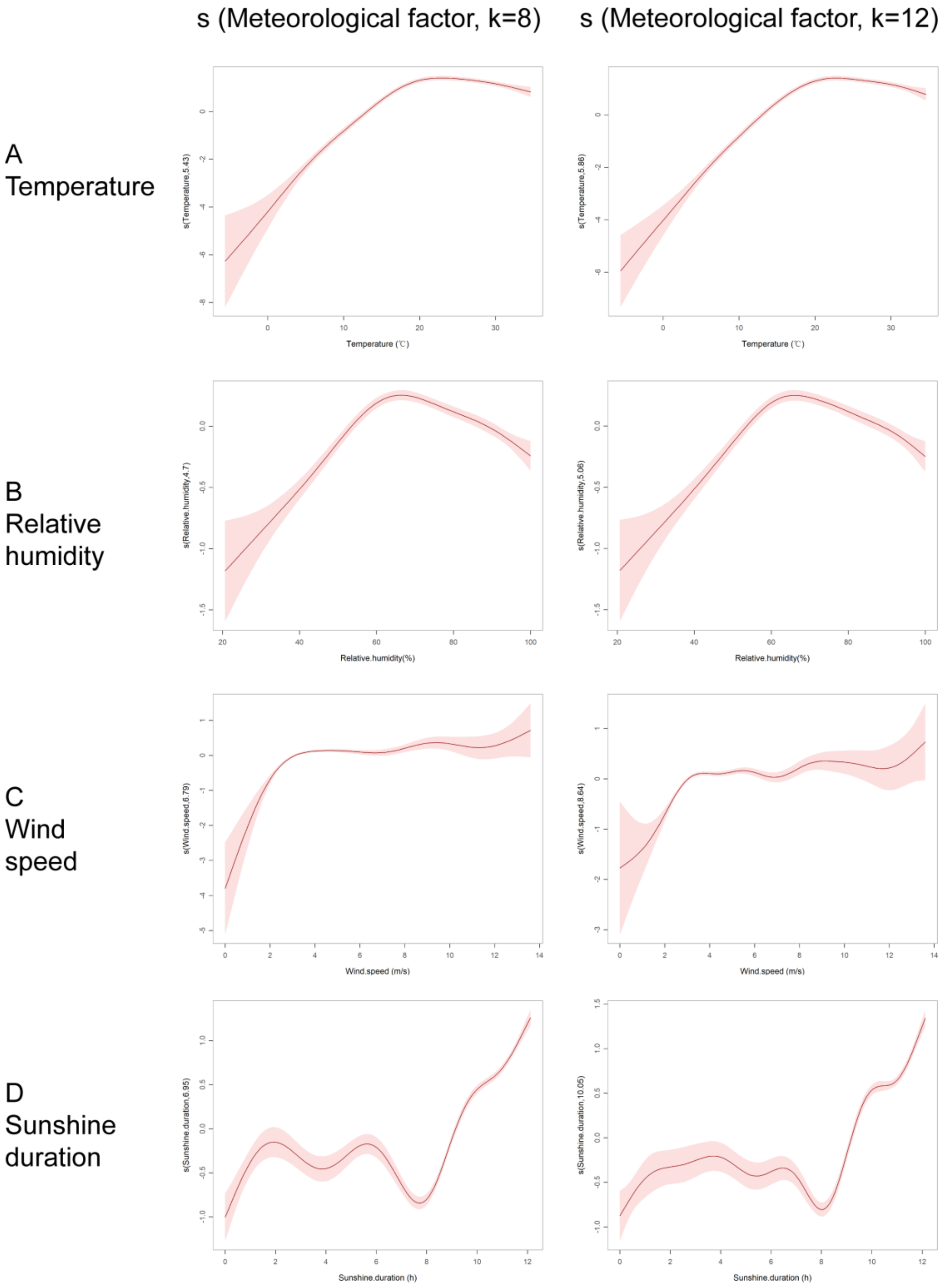

Figure S3 Sensitivity analysis: exposure-response curves for the effects of different meteorological factors on daily SFTS cases in the single-variable model using univariate GAM at different degree of freedom (k=8 / 12). A (Daily mean temperature); B (Daily mean atmospheric pressure); C (Daily mean relative humidity); D (Daily mean wind speed); E (Daily sunshine duration).

Note: The x-axis is the meteorological parameters. The y-axis indicates the contribution of the smoother to the fitted value. A Y-axis value of 0 implies that the contribution of meteorological factors to the number of SFTS cases is at the baseline level. A Y-axis value >

0 indicates a positive contribution of meteorological factors to the number of SFTS cases, meaning an increased risk of incidence. Conversely, a Y-axis value < 0 indicates the opposite situation. S (Temperature, 5.43), where 5.43 represents the effective degree of freedom (EDF) of the smoothing function.

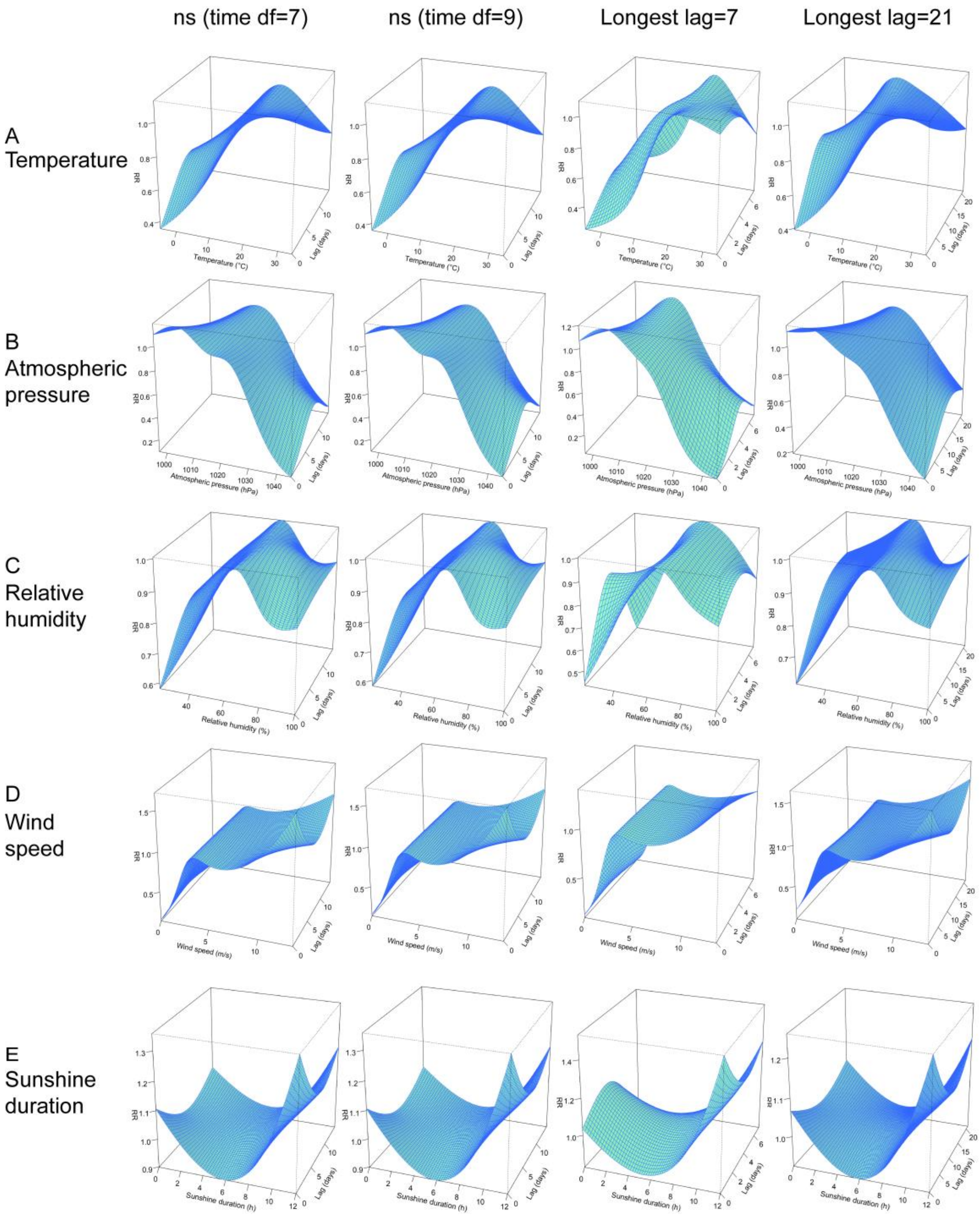

Figure S4 Sensitivity analysis: the estimated exposure-lag-response 3D graphs of meteorological factors at different ns time (7 /9) and longest lag days (7 /21). A (Daily mean temperature); B (Daily mean atmospheric pressure); C (Daily mean relative humidity); D (Daily mean wind speed); E (Daily sunshine duration)

Table S1 Summary statistics of the SFTS cases in Xinyang from 2013 to 2023

Case	data	Case - year	number	Case - year	number
Case – maximum / day	0	Total	6601	2019	298
Case - minimum / day	19	2013	610	2020	476
Case - mean / day	1.64	2014	789	2021	381
Male / female	0.69	2015	1028	2022	485
Age - maximum	93	2016	868	2023	773
Age - minimum	1	2017	576		
Age - mean	61.52	2018	317		

Note: SFTS severe fever with thrombocytopenia syndrome

Table S2 Summary statistics of the meteorological factors in Xinyang from 2013 to 2023

Variable	Unit	Mean	SD	Min	P25	P50	P75	Max
Mean temperature	℃	16.91	9.35	-5.60	9.00	17.70	24.90	34.60
Mean atmospheric pressure	hPa	1016.49	9.98	996.10	1007.50	1016.75	1024.30	1045.20
Mean relative humidity	%	68.36	17.66	20.70	55.30	69.20	82.10	100.00
Mean wind speed	m/s	4.10	1.58	0.00	2.90	3.90	4.900	13.60
Mean Sunshine duration	h	8.51	2.72	0.00	7.76	9.00	10.7	12.12

Note: SFTS severe fever with thrombocytopenia syndrome

Table S3 Model test of the interaction analysis using multivariable GAM

Variable	Edf	Ref.df	Chi.sq	P-value
Mean temperature	14.00	10.03	48.46	<0.001
Mean relative humidity	1.24	1.44	4.69	0.089
Mean wind speed	5.01	6.05	5.74	0.477
Mean sunshine duration	6.28	7.42	19.75	0.010
“Mean temperature” “Mean relative humidity”	13.71	19.00	69.43	<0.001
“Mean temperature” “Mean wind speed”	9.68	13.40	51.75	<0.001
“Mean temperature” “Sunshine duration”	10.14	14.00	51.15	<0.001

Table S4 Interpretation of the model (McFadden' R^2) by DNLM model covariates "time" and "DOW"

Variable	Full model (McFadden' R^2)	Reduced model (McFadden'R^2)	ΔR^2	F	P value
Mean temperature	0.4970	0.4883	0.0087	1.609	0.012
Mean atmospheric pressure	0.5102	0.5012	0.0090	1.363	0.073
Mean relative humidity	0.0977	0.0887	0.0090	0.936	0.585
Mean wind speed	0.1525	0.1435	0.0090	0.871	0.689
Mean sunshine duration	0.3017	0.2928	0.0089	1.213	0.179

Note: Full model include covariates "time" and "DOW"; Reduced model does not include covariates "time" and "DOW"

Table S5 The highest cumulative RR and 95% CI between daily meteorological factors and the SFTS incidence at lag 0-14 days

Variable	Lag (day)	Unit	Value	Cum RR	95%CI-low	95%CI-high
Mean temperature	0-14	℃	21	1.255	1.181	1.333
Mean atmospheric pressure	0-14	hPa	1010	1.655	1.431	1.915
Mean relative humidity	0-14	%	68	1.008	0.992	1.025
Mean wind speed	0-14	m/s	13.4	14.836	1.940	113.453
Sunshine duration	0-14	h	12	13.674	11.185	16.717

Table S6 The RR and 95% CI between daily mean temperature at the 5th (1.7℃) and 95th (30.4℃) and SFTS across lag 0-14 days

Variable	Lag (day)	RR P5	95%CI-low P5	95%CI-high P5	RR P95	95%CI-low P95	95%CI-high P95
Mean temperature	0	0.491	0.373	0.646	1.124	1.062	1.189
Mean temperature	1	0.259	0.160	0.418	1.233	1.119	1.360
Mean temperature	2	0.147	0.079	0.273	1.322	1.167	1.498
Mean temperature	3	0.089	0.043	0.180	1.384	1.203	1.592
Mean temperature	4	0.057	0.026	0.122	1.415	1.222	1.639
Mean temperature	5	0.038	0.017	0.085	1.413	1.217	1.640
Mean temperature	6	0.027	0.012	0.061	1.378	1.186	1.601
Mean temperature	7	0.019	0.008	0.046	1.313	1.127	1.530
Mean temperature	8	0.014	0.006	0.035	1.222	1.044	1.430
Mean temperature	9	0.010	0.004	0.027	1.112	0.946	1.306
Mean temperature	10	0.008	0.003	0.020	0.988	0.841	1.160
Mean temperature	11	0.005	0.002	0.014	0.858	0.734	1.001
Mean temperature	12	0.004	0.001	0.010	0.728	0.631	0.839
Mean temperature	13	0.003	0.001	0.006	0.603	0.530	0.687
Mean temperature	14	0.002	0.001	0.004	0.489	0.429	0.556

Table S7 The RR and 95% CI between daily mean atmospheric pressure at the 5th (1002 hPa) and 95th (1032 hPa) and SFTS across lag 0-14 days

Variable	Lag (day)	RR P5	95%CI-low P5	95%CI-high P5	RR P95	95%CI-low P95	95%CI-high P95
Mean atmospheric pressure	0	1.168	1.098	1.242	0.547	0.434	0.688
Mean atmospheric pressure	1	1.330	1.195	1.480	0.311	0.207	0.467
Mean atmospheric pressure	2	1.477	1.288	1.693	0.184	0.108	0.314
Mean atmospheric pressure	3	1.599	1.370	1.866	0.113	0.061	0.210
Mean atmospheric pressure	4	1.688	1.435	1.986	0.071	0.036	0.141
Mean atmospheric pressure	5	1.739	1.475	2.050	0.046	0.022	0.096
Mean atmospheric pressure	6	1.747	1.480	2.063	0.030	0.014	0.065
Mean atmospheric pressure	7	1.714	1.448	2.028	0.020	0.009	0.045
Mean atmospheric pressure	8	1.640	1.380	1.949	0.013	0.006	0.031
Mean atmospheric pressure	9	1.532	1.285	1.827	0.009	0.004	0.021
Mean atmospheric pressure	10	1.398	1.174	1.665	0.005	0.002	0.014
Mean atmospheric pressure	11	1.246	1.055	1.471	0.003	0.001	0.008
Mean atmospheric pressure	12	1.084	0.933	1.260	0.002	0.001	0.005
Mean atmospheric pressure	13	0.922	0.807	1.053	0.001	0.000	0.003
Mean atmospheric pressure	14	0.765	0.672	0.872	0.001	0.000	0.002

Table S8 The RR and 95% CI between daily mean relative humidity at the 5th (38.2%) and 95th (95.7%) and SFTS across lag 0-14 days

Variable	Lag (day)	RR P5	95%CI-low P5	95%CI-high P5	RR P95	95%CI-low P95	95%CI-high P95
Mean relative humidity	0	0.730	0.669	0.796	0.847	0.786	0.912
Mean relative humidity	1	0.549	0.471	0.640	0.727	0.638	0.829
Mean relative humidity	2	0.426	0.349	0.520	0.633	0.532	0.752
Mean relative humidity	3	0.340	0.270	0.428	0.557	0.456	0.681
Mean relative humidity	4	0.279	0.217	0.358	0.496	0.398	0.618
Mean relative humidity	5	0.233	0.179	0.304	0.446	0.353	0.563
Mean relative humidity	6	0.199	0.152	0.262	0.403	0.315	0.516
Mean relative humidity	7	0.173	0.130	0.230	0.366	0.283	0.475
Mean relative humidity	8	0.151	0.112	0.203	0.333	0.253	0.439
Mean relative humidity	9	0.132	0.096	0.180	0.303	0.227	0.405
Mean relative humidity	10	0.115	0.083	0.159	0.275	0.203	0.371
Mean relative humidity	11	0.100	0.072	0.139	0.247	0.181	0.338
Mean relative humidity	12	0.087	0.062	0.121	0.222	0.161	0.304
Mean relative humidity	13	0.074	0.053	0.104	0.197	0.142	0.273
Mean relative humidity	14	0.063	0.045	0.090	0.174	0.123	0.246

Table S9 The RR and 95% CI between daily mean wind speed at the 5th (2.2 m/s) and 95th (7.3 m/s) and SFTS across lag 0-14 days

Variable	Lag (day)	RR P5	95%CI-low P5	95%CI-high P5	RR P95	95%CI-low P95	95%CI-high P95
Mean wind speed	0	0.711	0.636	0.795	0.939	0.856	1.030
Mean wind speed	1	0.511	0.420	0.623	0.878	0.745	1.035
Mean wind speed	2	0.372	0.286	0.483	0.818	0.657	1.019
Mean wind speed	3	0.273	0.201	0.371	0.760	0.587	0.985
Mean wind speed	4	0.201	0.143	0.283	0.705	0.528	0.942
Mean wind speed	5	0.149	0.104	0.215	0.653	0.477	0.895
Mean wind speed	6	0.111	0.075	0.164	0.605	0.431	0.850
Mean wind speed	7	0.082	0.054	0.124	0.563	0.391	0.810
Mean wind speed	8	0.060	0.038	0.093	0.525	0.355	0.777
Mean wind speed	9	0.043	0.027	0.069	0.494	0.326	0.749
Mean wind speed	10	0.031	0.019	0.050	0.468	0.302	0.726
Mean wind speed	11	0.021	0.013	0.036	0.448	0.284	0.706
Mean wind speed	12	0.014	0.009	0.024	0.433	0.270	0.693
Mean wind speed	13	0.010	0.006	0.017	0.423	0.260	0.688
Mean wind speed	14	0.006	0.003	0.011	0.939	0.856	1.030

Table S10 The RR and 95% CI between daily sunshine duration at the 5th (2.16 h) and 95th (11.5 h) and SFTS across lag 0-14 days

Variable	Lag (day)	RR P5	95%CI-low P5	95%CI-high P5	RR P95	95%CI-low P95	95%CI-high P95
Sunshine duration	0	0.990	0.912	1.075	1.265	1.211	1.321
Sunshine duration	1	0.974	0.842	1.127	1.559	1.445	1.682
Sunshine duration	2	0.954	0.787	1.157	1.872	1.696	2.067
Sunshine duration	3	0.931	0.743	1.166	2.196	1.961	2.460
Sunshine duration	4	0.904	0.704	1.160	2.521	2.232	2.849
Sunshine duration	5	0.875	0.669	1.144	2.843	2.503	3.229
Sunshine duration	6	0.846	0.635	1.126	3.160	2.770	3.604
Sunshine duration	7	0.817	0.602	1.108	3.480	3.036	3.989
Sunshine duration	8	0.789	0.570	1.093	3.816	3.311	4.399
Sunshine duration	9	0.764	0.541	1.080	4.189	3.616	4.853
Sunshine duration	10	0.742	0.516	1.068	4.620	3.977	5.368
Sunshine duration	11	0.724	0.496	1.057	5.137	4.422	5.967
Sunshine duration	12	0.709	0.480	1.049	5.771	4.980	6.687
Sunshine duration	13	0.699	0.465	1.049	6.562	5.672	7.591
Sunshine duration	14	0.692	0.449	1.065	7.557	6.495	8.793
